# Clinical application of real-time PET/CT guided targeted retroperitoneal masses biopsy in diagnosing malignant tumors

**DOI:** 10.1186/s12885-023-11334-y

**Published:** 2023-09-05

**Authors:** Xiaomin Li, Wanchun Zhang

**Affiliations:** 1 Tongji Shanxi Hospital, Shanxi Bethune Hospital, Shanxi Academy of Medical Sciences, Third Hospital of Shanxi Medical University, Longcheng Street NO.99, 030032 Taiyuan, China; 2 Tongji Hospital, Tongji Medical College, Huazhong University of Science and Technology, Wuhan, 430030 China

**Keywords:** Retroperitoneal mass, Biopsy, PET/CT, 18F-FDG

## Abstract

**Objective:**

To explore the feasibility, safety, and clinical application value based on the fusion image of 18 F-FDG PET/CT, for guiding retroperitoneal puncture biopsy technology and to determine the diagnosis of retroperitoneal masses in diagnosing malignant tumors.

**Methods:**

From March 2019 to January 2023, 42 patients underwent 18 F-FDG PET/CT imaging and were found to have retroperitoneal lesions that required definite diagnosis; 22 were male, 20 were female, and the average age was(59.17 ± 13.23) years. According to the fused 18 F-FDG PET/CT tomographic image, the target point with the highest metabolic activity, the safest, and expected maximum sample size was selected. CT scans were acquired with the same machine and fused with 18 F-FDG PET, guiding the puncture biopsy needle to approach the expected target zone, enabling timely delivery of pathological and immunohistochemical examination of the biopsy. Success rate, total examination time, biopsy operation time, complications, CT radiation dose, pathological, and immunohistochemical results were recorded.

**Results:**

All 42 patients were sampled successfully with the successful rate being 100%. The site of sampling of 42 patients accurately targeted the highest metabolic activity, the safest, and the expected maximum sample size. All 42 patients received clear diagnosis (25 cases of malignant tumors and cases of 17 benign tissues). 15 cases of patients had a change in clinical diagnosis, accounting for 35.7% of all patients, and affecting subsequent treatment plans. The average total examination time for patients was (41.3 ± 7.3) minutes, and the biopsy operation time was (29.1 ± 8.7) minutes. The effective radiation dose generated by the entire examination generated by CT guidance was (2.0 ± 0.5) mSv; no severe complications occurred in the patients.

**Conclusion:**

Real-time-guided retroperitoneal puncture biopsy based on 18 F-FDG PET/CT fusion image is safe, accurate, and feasible, and can provide patients of retroperitoneal mass with clear pathological diagnosis and immunohistochemical evaluation.

## Background

Retroperitoneal tumors refer to tumors that originate in the retroperitoneal space (including the presacral and pelvic floor spaces, outside the solid organs of the retroperitoneum), with an incidence rate of (0.5 ~ 1.0)/100,000. The retroperitoneal space is anatomically deep, the types of tumor tissue are diverse, and the clinical manifestations lack specificity. Patients often do not have obvious clinical symptoms in the early stages, leading to diagnosis at an advanced stage. Tumors extensively infiltrate surrounding tissues, making them prone to recurrence and metastasis after surgery. Therefore, there are significant challenges in the diagnosis and treatment of retroperitoneal tumors [1].

Computed tomography (CT) and magnetic resonance imaging (MRI) play important roles in the differentiation of benign and malignant retroperitoneal lesions. Regardless, it remains difficult to differentiate masses in the retroperitoneum given their heterogeneity and the substantial overlap of imaging findings [2, 3].

Positron emission tomography/computed tomography (PET/CT) is a diagnostic tool that allows tissue metabolism and perfusion evaluation, and 2-[18F]-fluoro-2-deoxy-D-glucose ([18F]FDG) is the most widely used and currently preferred radiotracer [4, 5]. This tracer allows for differentiation between normal and cancerous tissue, considering that neoplastic cells have increased glucose metabolic activity [6–8], but false positive findings due to inflammatory processes (especially granulomatous) are frequent. There have been few previous studies showing the degree of FDG uptake according to the histological tumour type of retroperitoneal masses. FDG uptake was correlated with the tumour grade. Diagnosing a retroperitoneal mass based solely on FDG uptake may lead to misdiagnosis [9]. Dao-ning Liu et al drawed the conclusion that 18F-FDG-PET/CT cannot simply distinguish malignant and benign tumors from the observation of retroperitoneal/intra-abdominal Tumors, and the SUVmax of malignant tumors, and inflammatory pseudotumor is higher than the SUVmax of benign tumors, lymph node metastasis, hematoma, and low malignant soft tissue sarcomas group. Guidance of“SUVmax location” may be helpful for biopsy and pathology dissection [10].

Imaging examinations have important clinical value in the diagnosis and treatment of retroperitoneal tumors. However, biopsy remains the “gold standard” for the diagnosis of retroperitoneal masses. The Chinese consensus on the diagnosis and treatment of retroperitoneal tumors (2019 version) recommends that if the tumor is difficult to remove based on imaging evaluation or differential diagnosis is needed, ultrasound or CT guided core needle biopsy is strongly recommended. To obtain sufficient tissue samples for pathological and molecular testing, it is recommended to perform biopsy in the area with a high standardized uptake value in PET-CT. Experienced physicians are recommended to perform retroperitoneal masses biopsy (RMB) [11].

However, due to the lack of professional skills and special techniques, and the inability of routine imaging to confirm high metabolic sites, clinical practice cannot be carried out routinely. Nuclear medicine molecular imaging has unique advantages in target area selection because it can simultaneously display anatomical and metabolic information [12, 13]. After mastering PET/CT-guided percutaneous biopsy technique, the author applied this technique to retroperitoneal mass biopsy to clarify the pathological diagnosis and evaluate its feasibility and clinical value. The results are reported as follows:

## Methods

### Patients

Clinical Data A retrospective selection of 42 patients who underwent 18 F-FDG PET/CT imaging between March 2019 and January 2023 was reviewed to clarify the diagnosis of retroperitoneal lesions. There were 22 male and 20 female patients with an average age of (59.17 ± 13.23) years. Clinical diagnoses included colon cancer in 6 patients, high-grade serous ovarian carcinoma in 4 patients, lymphoma in 7 patients, tuberculosis in 5 patients, retroperitoneal fibrosis in 11 patients, and 9 undiagnosed cases. (Table 1) Due to clinical suspicion of retroperitoneal fibrosis and the inability to differentiate it from primary or metastatic malignancies, abnormal metabolic foci in the retroperitoneum were discovered by 18 F-FDG PET/CT imaging, and percutaneous biopsy was required for histological confirmation. Before biopsy, all patients underwent coagulation series and platelet count tests to ensure no significant abnormalities in coagulation, platelet count, bleeding time, and prothrombin time, and to exclude patients who cannot cooperate with the procedure.

**Table 1:** The patient’ characteristics

Characteristics	n (%)
Age(years)	
Range	22 ~ 80
Mean Sex Male Female Clinical diagnosis Malignant High-grade serous ovarian carcinoma Colon cancer Lymphoma Retroperitoneal fibrosis Benign Tuberculosis Undiagnosed cases	59.17 ± 13.23 22 (52.4%) 20 (47.6%) 4 (9.5%) 6 (14.3%) 7 (16.7%) 11 (26.2%) 5 (11.9%) 9 (21.4%)

### 18 F-FDG PET/CT technique and imaging

The imaging agent used was 18 F-FDG, and the cyclotron accelerator used was the GE MINItrace Qilin system. The FDG precursor drug box was purchased from ABX Limited and prepared using the 18O (p, n) 18 F nucleophilic reaction with a radiochemical purity requirement of > 95%. Imaging was performed using the GE Discovery PET/CT Elite system. Patients fasted for more than 6 h before examination and had a fasting blood glucose level of less than 10mmol. The imaging agent was injected intravenously at a dose of 4.44MBq/kg according to body weight, and images were collected 45–60 min after injection. CT acquisition parameters: free breathing, tube voltage of 140 kV, current of 300mA, bed speed of 48.43 mm/s, and slice thickness of 5 mm. PET acquisition parameters: PET was performed with a 15.7 cm bed for each bed position, with a scanning time of 1.5 min. The scanning range was from the head to the upper middle of the thigh, and the energy peak was 511KeV. The matrix was 192 × 192, the magnification was 1.0, and CT data was used for attenuation correction. VUE point HD was used for iterative reconstruction to obtain cross-sectional, sagittal, and coronal CT, PET, and PET/CT fusion images.

### Image analysis

PET-CT images were analyzed independently by two experienced nuclear medicine physicians on workstation computers using Xeleris software. Experienced nuclear medicine physicians are defined as those with more than ten years of experience in diagnostic PET/CT imaging.

The readers were blinded to patients’ clinical information including previous therapy and the findings of other imaging modalities. In case of any discrepancy regarding the findings of PET-CT images, a consensus was reached after mutual discussion. The site and characters of the lesions were also identified.

### Image analysis and procedure for percutaneous biopsy

After the ^18^ F-FDG PET/CT fusion tomography was completed, the highest metabolic activity and safest location with the largest expected sample volume were selected as the sampling target based on the fusion tomography image. A puncture plan was formulated to determine patient position, needle entry point, angle, and depth.

The 18 F-FDG PET/CT biopsy procedure was guided using the same PET/CT scanner in same day or within one week after the day of completion of 18 F-FDG PET/CT fusion tomography imaging, and the needle tip position was obtained using previously acquired PET images fused with intraoperative CT.

Percutaneous biopsy procedure: The basic process of the biopsy was explained to the patient and family members, and the purpose, risk, and complications of the biopsy were described. The patient’s cooperation was obtained, and an informed consent form was signed. Routine tests of coagulation function, blood routine, blood biochemistry, and serology (hepatitis B, hepatitis C, HIV, and syphilis) were performed before surgery, as well as an abdominal enhanced CT. The disinfection room was set up, and the patient’s temperature, respiration, blood pressure, pulse, and cardiopulmonary function were checked. The Bard 1816 A disposable biopsy needle and the matching coaxial biopsy cannula needle were used. First, CT localization scanning was performed (voltage of 120 kV, current of 20 mA, and scanning length of 50 cm), and the homemade metal grid was placed on the surface of the skin in the puncture area. The puncture point was marked using the laser positioning line and metal grid marked by CT, and the puncture angle and depth were determined. Subsequently, a low-dose chest CT scan was performed (conditions: 120 kV, 20 mA). After routine disinfection and draping, local anesthesia was performed using 1% lidocaine, and the needle was gradually inserted according to the predetermined position and depth. The 1816 A Bard coaxial biopsy needle was used to stepwise insert into the biopsy target area, and CT scan was used to fuse with 18 F-FDG PET through the built-in VMI software to confirm that the needle tip was located in the most metabolically active area. The needle core was withdrawn, and the automatic biopsy gun was inserted and fired to obtain tissue samples. After the biopsy, the biopsy tissue was examined, and if the biopsy was satisfactory, it was placed in a 10% formalin solution for fixation and sent to the pathology department for histological and immunohistochemical testing, and the operation was completed. If the biopsy was unsatisfactory, it could be repeated once or twice depending on the patient’s condition. The puncture site was disinfected and compressed for 5 min to stop bleeding. After no oozing, the site was disinfected and bandaged. The patient was observed in the recovery room for 30 min, and could be discharged if there were no adverse reactions. The success rate of the biopsy, total examination time, biopsy operation time, complications, CT radiation dose, and pathological and immunohistochemical results were recorded. All percutaneous biopsy operations under PET/CT guidance were performed by physicians in our department with more than 3 years of percutaneous biopsy experience.

### Follow-up and reference standard

All the previous clinical pathological data of 42 patients have been followed up as soon as possible and their molecular subtypes have been classified. We derived the final diagnoses from histopathology and clinical/imaging follow-up (CT, MRI, PET -CT) over at least 6 months.

### Statistical analysis and ethics

Postoperative records included total examination time (the time from the patient entering the PET/CT room to leaving), biopsy operation time (the time from formulating the sampling plan until completing the biopsy), complications, CT radiation dose, and biopsy success rate. The CT radiation dose received by the patient was expressed as the sum of the dose-length products (DLP, mGy) for each scan. The effective radiation dose was calculated as the DLP multiplied by the weighting factor κ (0.016 mSv•mGy-1•cm-1 for the abdomen and pelvis). Normally distributed metric data were expressed as x ± s. The pathological and immunohistochemical results of the follow-up biopsy patients were recorded. According to the the final diagnoses from histopathology and clinical/imaging follow-up, the diagnosis of PET/CT and PET/CT-guided RMB were classified as true-positive (TP), false-positive (FP), true-negative (TN), and false-negative (FN). Sensitivity, specificity, positive predictive value (PPV) and negative predictive value (NPV) were is calculated according to the number of TN, TP, FP, FN, and determined on the basis on number of patients, not number of lesions. McNemar test was used to test differences in the sensitivity and specificity between PET/CT and PET/CT-guided RMB. Chi-square test was used to test differences in the NPV and PPV between PET/CT and PET/CT-guided RMB. All statistical analysis was performed using SPSS, version 26 software. A p-value < 0.05 was considered significant. This retrospective evaluation of collected data was approved by the ethics committee of our institution. This study was approved by the Ethics Committee of our hospital (approval number: YXLL-2020-033).

## Results

### Puncture site and patient position

Of the 42 patients, 38 were in the prone position and 4 were in the lateral position. The puncture biopsy site was on the left retroperitoneum of the aorta in 23 cases and on the right retroperitoneum of the aorta in 19 cases. The target lesion was wrapped around the aorta in 15 cases, the left ureter in 8 cases, the right ureter in 7 cases, and the aorta and ureter in 12 cases. (Table 2)

**Table 2:** Sites and CT pattern of evaluated lesions

Vriable	n	%
Sites	42	100
The left retroperitoneum of the aorta	23	54.7
The right retroperitoneum of the aorta	19	45.3
Characters		
Around the aorta	15	35.7
Around the left ureter	8	19.0
Around the left ureter	7	16.7
Around the aorta and ureter	12	28.6

### Success rate and complications

**Figs. 1:**
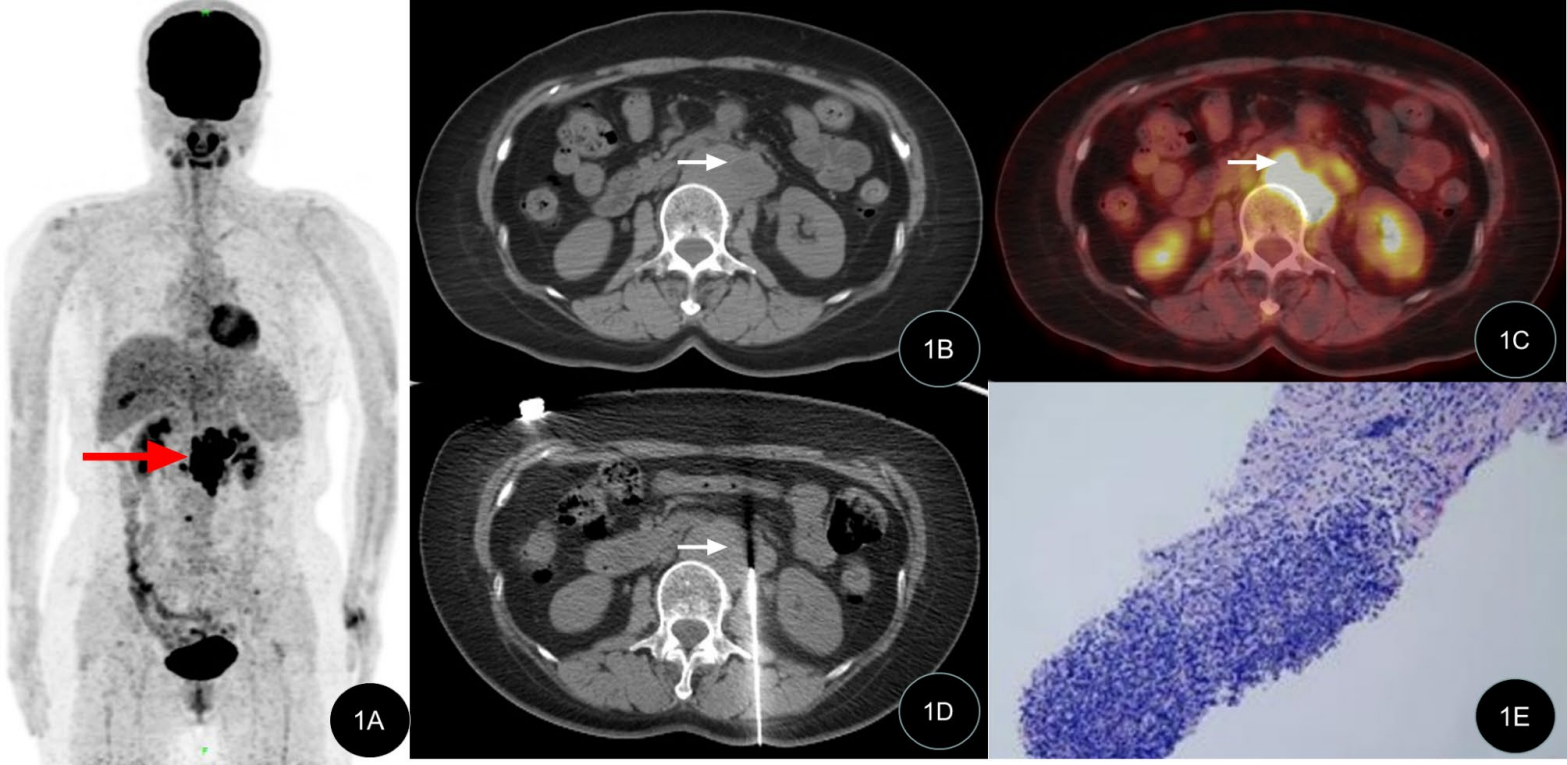
PET/CT-guided targeted retroperitoneal masses biopsy The patient (40-year-old male) was admitted due to abdominal pain. The PET/CT MIP image, PET/CT fusion CT image, puncture biopsy image, and biopsy pathological image are shown. 1 A. MIP image shows abnormal metabolism increase at the left side of the lumbar spine (indicated by red arrow); 1B, 1 C. PET/CT fusion CT image shows increased metabolism at the retroperitoneal mass; 1D. Puncture biopsy of the target area of the retroperitoneal mass under PET/CT guidance; 1E. Pathological examination shows highly invasive B-cell lymphoma (HE × 100)

**Figs. 2:**
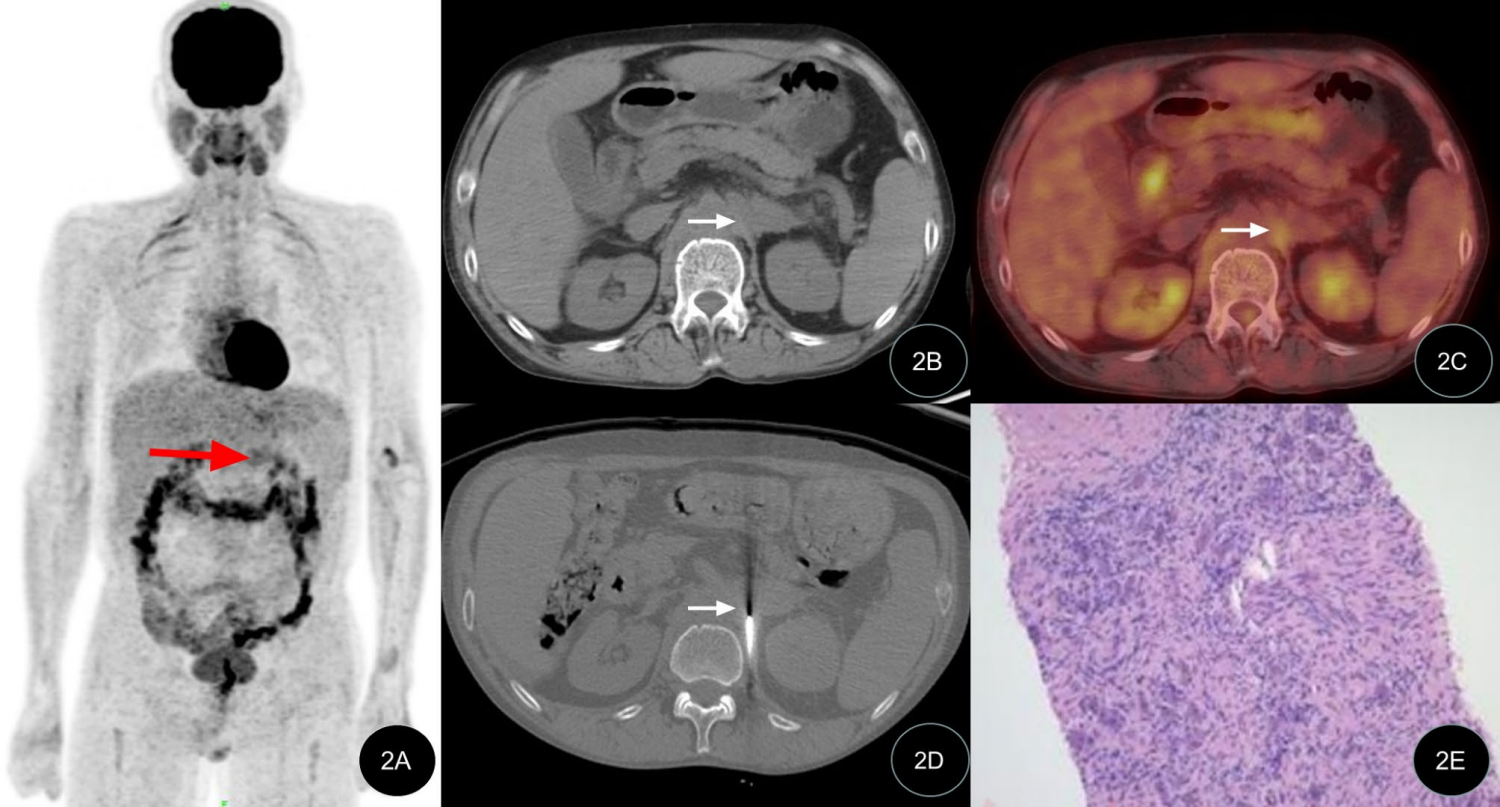
PET/CT-guided targeted retroperitoneal masses biopsy The patient (49-year-old male) was admitted due to abdominaldistension. The PET/CT MIP image, PET/CT fusion CT image, puncture biopsy image, and biopsy pathological image are shown. 2A MIP image shows abnormal metabolism increase at the left side of the lumbar spine (indicated by red arrow); 2B, 2C PET/CT fusion CT image shows increased metabolism at the retroperitoneal mass; 2D Puncture biopsy of the target area of the retroperitoneal mass under PET/CT guidance; 2E Pathological examination shows proliferative fibrotic tissue (HE × 100)

**Figs. 3:**
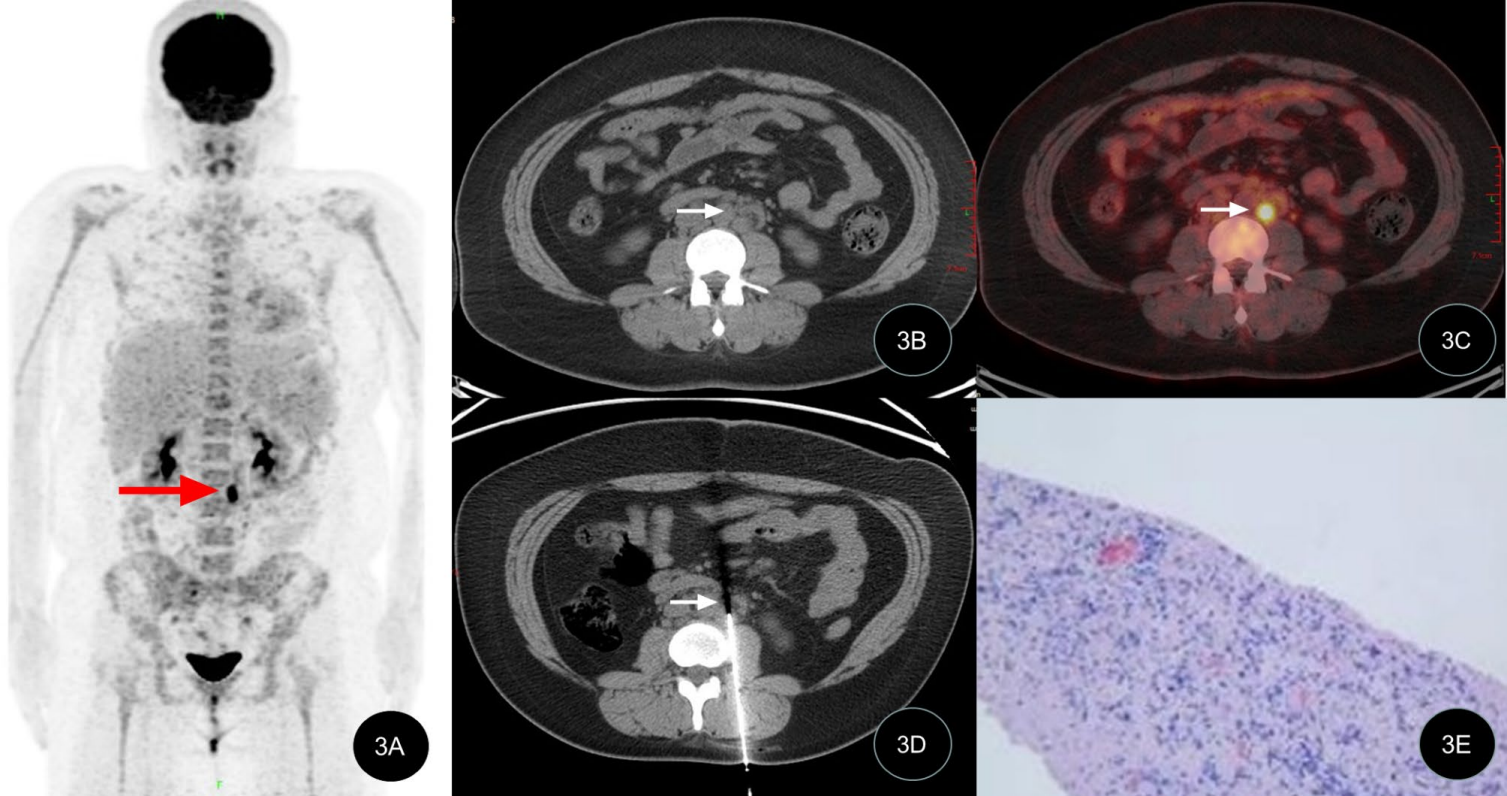
PET/CT-guided targeted retroperitoneal masses biopsy Lymphoproliferative disorders; The patient (22-year-old female) was admitted due to mild fever with abdominal pain. The PET/CT MIP image, PET/CT fusion CT image, puncture biopsy image, and biopsy pathological image are shown. 3A MIP image shows abnormal metabolism increase at the left side of the lumbar spine (indicated by red arrow); 3B, 3C PET/CT fusion CT image shows increased metabolism at the retroperitoneal lymph node; 3D Puncture biopsy of the target area of the retroperitoneal lymph node under PET/CT guidance; 3E Pathological examination shows kikuchi disease (HE × 100)

All 42 patients successfully underwent percutaneous biopsy guided by CT, with a 100% success rate. A total of 42 specimens were obtained and subjected to pathological examination, with an average total examination time of (41.3 ± 7.3) min and biopsy operation time of (29.1 ± 8.7) min. The effective radiation dose produced by the CT-guided operation during the entire examination process was (2.0 ± 0.5) mSv. No serious complications occurred in any of the patients, and no significant bleeding or nerve damage was found after the operation. Of the 42 patients, 28 cases were satisfied with the first biopsy tissue, 10 cases underwent a second sampling and 4 cases underwent three samplings due to the low amount of tissue in the first procedure. All specimens met the requirements of pathological histological examination. Typical case images are shown in Figs. 1, 2 and 3.

### Diagnostic performance of PET/CT vs. PET/CT-guided RMB

All patients underwent at least 6 months of imaging follow-up and there were no lost patients. According to the pathological results and follow-up results, malignant tumors were confirmed in 25 of the 42 patients (59%). PET/CT was positive for disease in 20, yielding a sensitivity of 80% (20/25). Because of 1 case was false negative, the sensitivity of PET/CT-guided RMB was 96% (24/25). PET/CT-guided RMB exhibited significantly higher sensitivity when compared to PET/CT for determination (p = 0.025 < 0.05). (Table 3)

**Table 3:** Sensitivity, specificity, positive predictive value, negative predictive value, and accuracy of PET/CT, and PET/CT-guided targeted retroperitoneal masses biopsy in diagnosing malignant tumors

Parameter	PET/CT	PET/CT-guided RMB	P value
Sensitivity	80	96	0.025*
Specificity	76	100	
PPV	79	95	
NPV	77	94	0.028*
Accuracy	76	96.5	

CT, computed tomography; NPV, negative predictive value; PPV, positive predictive value;

PET/CT was true negative in 13 of 17 patients, yielding a specificity of 76% (13/17), whereas the specificity was of PET/CT-guided RMB (17/17,100%). (Table 3)

The PPV and NPV of PET/CT were 79% (19/24) and 77% (17/22) respectively, while PPV and NPV of PET/CT-guided RMB were 95% (19/20) and 94% (14/15) respectively. PET/CT-guided RMB demonstrated significantly higher PPVand NPV when compared to PET/CT (p = 0.028 < 0.05). (Table 3)

### Pathological diagnosis analysis

The pathological results of the 42 successfully sampled patients were as follows: 9 cases were proliferative fibroblastic tissue or fibrous tissue, 8 cases were chronic granulomatous lesions, 12 cases were lymphomas (3 cases of high-invasive B-cell lymphoma, 3 cases of B-cell non-Hodgkin lymphoma, 6 cases of marginal zone lymphoma). 13 cases were metastatic adenocarcinomas (8 cases of gastrointestinal origin and 5 cases of ovarian high-grade plasma adenocarcinoma origin); immunohistochemistry was performed in 25 patients with pathology suggestive of lymphoma or metastatic carcinoma. (Table 3)

## Discussion

The incidence of retroperitoneal tumors is (0.5-1.0)/100,000, and due to the deep location, retroperitoneal tumors are often discovered in the middle and late stages. The diagnosis and treatment of retroperitoneal tumors present great challenges. Imaging examination plays an important role in the diagnosis and treatment of retroperitoneal tumors, but biopsy remains the “gold standard” for diagnosing retroperitoneal masses.

The China Consensus on the Diagnosis and Treatment of Retroperitoneal Tumors (2019 edition) recommends that if the tumor is difficult to remove based on imaging evaluation or if differential diagnosis is needed, it is strongly recommended to perform a biopsy under imaging guidance.

In order to obtain sufficient tissue samples for pathological histological diagnosis and possible molecular detection, it is recommended to perform a biopsy in areas with high standardized uptake values on PET-CT. Therefore, biopsy has significant significance for the pathological diagnosis of retroperitoneal masses. Currently, the main method of biopsy guidance is conventional imaging guidance [14]. However, there are shortcomings in conventional imaging-guided biopsy [15]: (1) It is difficult to determine whether the lesion shown on conventional imaging has metabolism and whether it is a suspicious lesion; (2) Without obtaining whole-body staging information, it is impossible to select the lesion with the highest surgical safety for sampling biopsy [16].

Compared with conventional imaging, nuclear medicine 18 F-FDG PET/CT molecular imaging can display both anatomical and metabolic information and can simultaneously reflect metabolic status and local anatomical structure of lesions [12, 17]. Targeted biopsy guided by 18 F-FDG PET/CT can theoretically improve the accuracy and success rate of biopsy. Several studies have used 18 F-FDG PET/CT images to guide target point selection for biopsy, and some studies have used real-time 18 F-FDG PET/CT guidance, showing higher biopsy success rate and safety [18–21]. Many studies have shown that PET/CT-guided or guided biopsy can optimize the diagnostic rate of image-guided interventions [22–24].

Wei et al. provided a new integrated accurate re-biopsy algorithm for percutaneous FDG high metabolism target tissue biopsy under PET/CT guidance, which can improve precise individualized treatment and prolong survival [25]. Previous studies have also confirmed that this is an effective and safe method for evaluating high metabolism bone lesions in suspected advanced lung cancer patients [22]. Bing et al. concluded that targeted BMB under PET/CT guidance can supplement possible false positive PET/CT and false negative iliac crest biopsy results for the evaluation of bone marrow involvement in new lymphoma patients [19]. Rajender Kumar et al. confirmed that ARA-assisted ^18^ F-FDG PET/CT-guided percutaneous real-time biopsy of metabolically active abdominal and pelvic lesions is a technically feasible, safe and accurate method for pathological diagnosis. PET-guided biopsies were highly practical and useful in patients, especially in those with a previous inconclusive biopsy [26]. Our previous study proved that SPECT/CT-guided Bone marrow biopsy was safe, accurate, and feasible and significantly higher sensitivity and NPV when compared to SPECT/CT for detection of bone metastases in breast cancer patients [13].

This study applied 18 F-FDG PET/CT to real-time guided biopsy of retroperitoneal masses, and the results showed a high biopsy success rate (100%), good safety (no severe complications occurred), and small radiation dose (2.0 ± 0.5mSv). In this study, all 42 cases of patients succeeded in biopsy (100%). During PET/CT-guided biopsy, because all patients were imaged with 18 F-FDG PET/CT, the additional radiation dose only came from the positioning CT. Due to the advantages of PET/CT fusion images, the tube current and tube voltage in this study were only 120mV and 20mA, respectively, with an effective radiation dose of about (2.0 ± 0.5)mSv, which is lower than the dose of a chest CT scan and lower than the reported dose in the literature [27].

CT-guided percutaneous biopsy has the advantages of high diagnostic accuracy and low complications, and the main complications are bleeding and nerve damage. In this study, all 42 patients did not experience severe complications, mainly due to (1) comprehensive evaluation of retroperitoneal lesions by 18 F-FDG PET/CT, which can avoid selecting high-risk puncture sites and select the safest puncture sites for biopsy; (2) strict mastery of indications and contraindications; (3) selection of biopsy needles that are suitable, and operating skillfully and cautiously.

All 42 patients in this study obtained a clear diagnosis (25 cases of malignant tumors and cases of 17 benign tissues). 15 patients had a change in clinical diagnosis, accounting for 35.7% (15/42) of all patients, which affected the subsequent treatment plan.

## Conclusion

It is insufficient to evaluate retroperitoneal masses using 18 F-FDG PET/CT imaging. It is recommended that PET/CT-guided RMB is recommended if positive lesions were detected on PET/CT imaging, which has significantly higher sensitivity and NPV in detecting patients with retroperitoneal lesions compared to PET/CT.

The integrated approach that includes PET/CT and PET/CT-guided RMB, which can be performed in one stop in nuclear medicine department, can offer important opportunities for precision treatment and improved quality of life of patients with retroperitoneal masses.

In conclusion, this study suggests that 18 F-FDG PET/CT-guided retroperitoneal biopsy is safe, feasible, and does not significantly increase radiation dose. It provides patients with an opportunity for precise pathological diagnosis of retroperitoneal lesions and has great clinical value, and is worthy of clinical promotion and application.

## Data Availability

All data generated or analysed during this study are included in this published article.
